# Correction: The barley lectin, horcolin, binds high-mannose glycans in a multivalent fashion, enabling high-affinity, specific inhibition of cellular HIV infection

**DOI:** 10.1016/j.jbc.2021.101158

**Published:** 2021-09-03

**Authors:** Nisha Grandhi Jayaprakash, Amrita Singh, Rahul Vivek, Shivender Yadav, Sanmoy Pathak, Jay Trivedi, Narayanaswamy Jayaraman, Dipankar Nandi, Debashis Mitra, Avadhesha Surolia

For Fig. 3*A*, the schematic representation of the structure of Man7D3 was mistakenly duplicated with the one of Man5 during article preparation. The corrected images are presented in the revised Fig. 3*A*. In Table 1, there is an error in one of the exponential values. The error is only in the exponential term of Ka value and there is no change in Kd. This correction does not affect the results or the conclusions of the work. The authors apologize for inconvenience these errors may have caused readers.

**Figure:**
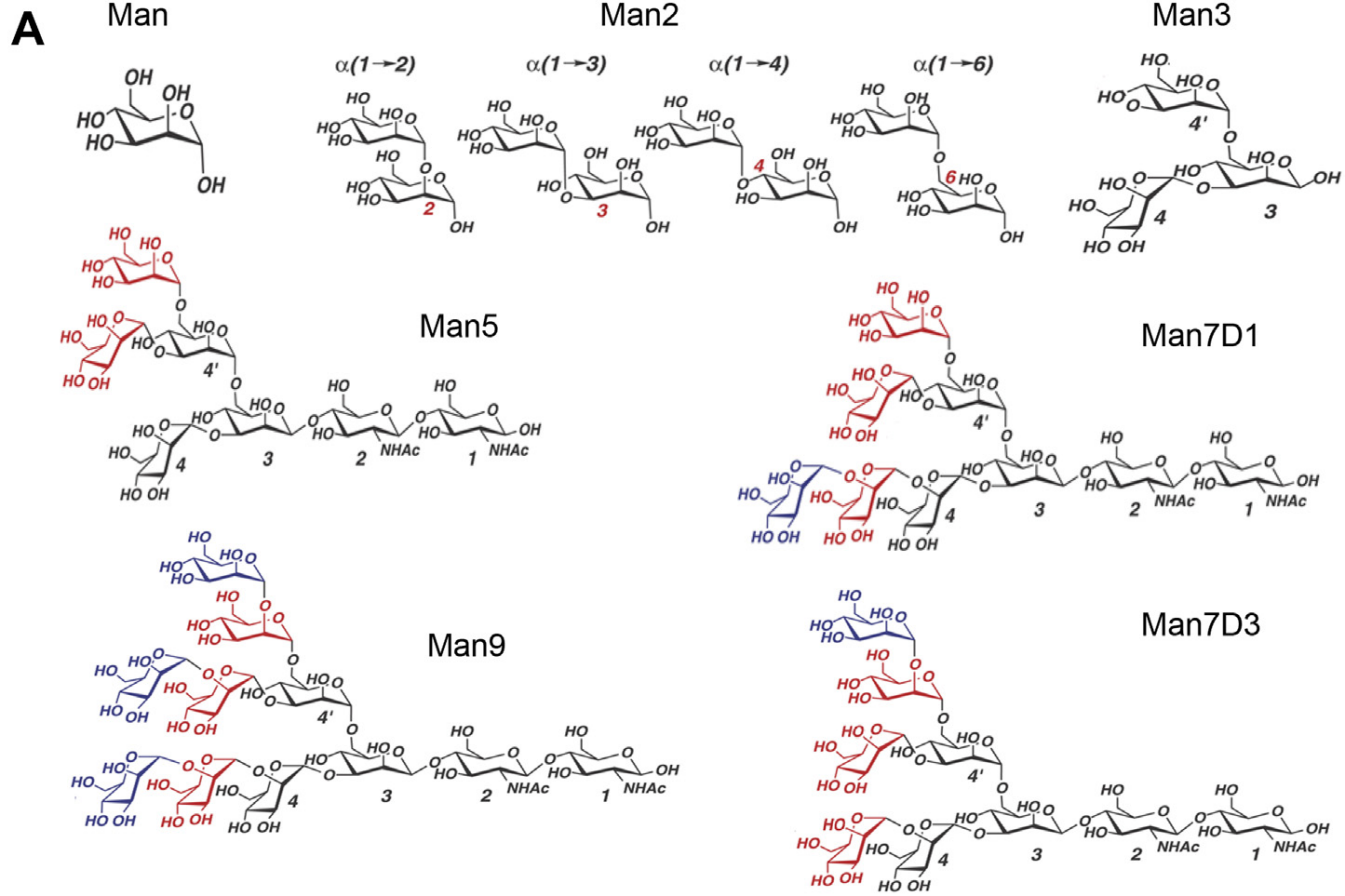

**Table:** Table 1. Thermodynamic parameters of manno-oligosaccharide binding to horcolin at 25 °C

Ligand	N	K_A_ (M^-1^)	K_D_	ΔH (kcal/mol)	ΔG (kcal/mol)	TΔS (kcal/mol)
Me-α-manno pyranoside	1.72 ± 0.328	200 ± 27.8	5.00 mM	–6.324	–3.1354	–3.1886
α-D-Man	1.90 ± 0.521	101 ± 12.5	9.90 mM	–5.517	–2.73368	–2.78332
Manα1-2-Man	1.946 ± 0.4028	398.9 ± 41.42	2.50 mM	–8.974	–3.5504	–5.4236
Manα-1-3-Man	1.88 ± 0.0724	596 ± 14.6	1.67 mM	–10.46	–3.7848	–6.6752
Manα-1-6-Man	1.78 ± 0.243	227 ± 10.3	4.40 mM	–14.88	–3.2282	–11.6518
Manα-1-4-Man	1.77 ± 0.649	196 ± 21.2	5.10 mM	–10.28	–3.128	–7.152
Man3	2.01 ± 0.0275	6.10 E3 ± 300	163 μM	–12.94	–5.1622	–7.7778
Man5	0.774 ± 0.076	5.99 E4 ± 1.00 E5	16.69 μM	–16.76	–6.5088	–10.2512
Man7-D1	0.925 ± 0.0117	2.61 E5 ± 2.87 E4	3.831 μM	–17.1	–7.3256	–9.7744
Man7-D3	1.09 ± 0.00711	2.4 E5 ± 1.45 E4	4.16 μM	–15.99	–7.348	–8.642
Man9	1.34 ± 0.018	8.60 E4 ± 1.21 E4	11.6 μM	–8.277	–6.73038	–1.54662

Abbreviations: N, stoichiometry coefficient (number of sugars bound per mole of horcolin monomer); ΔH, enthalpy; ΔS, entropy; ΔG, reaction energy (calculated with the formula ΔG = ‒RT ln K_b_, where R = 1.987 cal/mol_K).

